# Invertebrate Post-Segregation Distorters: A New Embryo-Killing Gene

**DOI:** 10.1371/journal.pbio.1001114

**Published:** 2011-07-26

**Authors:** Steven P. Sinkins

**Affiliations:** Department of Zoology, University of Oxford, Oxford, United Kingdom

## Abstract

Cytoplasmic incompatibility induced by inherited intracellular bacteria of arthropods, and *Medea* elements found in flour beetles, are both forms of postsegregation distortion involving the killing of embryos in order to increase the ratio of progeny that inherit them. The recently described *peel-zeel* element of *Caenorhabditis elegans* also uses this mechanism; like *Medea* the genes responsible are in the nuclear genome but it shares a paternal mode of action with the bacteria. The *peel-1* gene has now been shown to encode a potent toxin that is delivered by sperm, and rescued by zygotic transcription of the linked *zeel-1*. The predominance of self-fertilization in *C. elegans* has produced an unusual distribution pattern for a selfish genetic element; further population and functional studies will shed light on its evolution. The element might also have potential for use in disease control.

Selfish genetic elements, including meiotic drive genes, homing endonucleases, transposons, and B-chromosomes, employ a fascinating diversity of mechanisms to subvert of the laws of Mendelian segregation, illustrating the inherent vulnerability of genetic systems that have evolved to ensure the equal inheritance of maternal and paternal alleles [1],[2]. Postsegregation distorters achieve similar ends—a strong bias in their own favour—in a rather dramatic fashion: they cause the death of embryos that do not inherit any copies of the element. Two natural examples of embryo killer systems have long been known in invertebrates: cytoplasmic incompatibility induced by inherited bacteria [3]–[5], and *Medea* elements in flour beetles [6]; there are some striking parallels between these disparate systems.

*Wolbachia* and *Cardinium* are the only intracellular bacteria so far known to produce cytoplasmic incompatibility in arthropods; *Wolbachia* is a particularly widespread and common [7]. Their inheritance is solely from mother to egg, often at or close to 100% frequency (although in some species maternal transmission is much less efficient). Because males are a transmission dead-end, they can be freely manipulated. Sperm from *Wolbachia*-infected males is modified during maturation, prior to the loss of the bacteria themselves with the rest of the cytoplasm. When *Wolbachia*-infected sperm fertilize eggs from uninfected females, cell-cycle timing defects in the male pronucleus lead to developmental arrest, which usually immediately follows fertilization (the mechanism is assumed to be very similar for *Cardinium*) [8]–[11]. However, viable progeny are rescued when both parents carry the bacteria, as cell-cycle synchrony is restored. The consequence of this unidirectional incompatibility is that infected females have a strong selective advantage—they can mate with any males in the population, while uninfected females cannot. The strength of the driving force is initially relatively weak but increases quickly as the bacterial population frequency rises, allowing rapid spread—as has been directly observed in nature for *Wolbachia* in *Drosophila simulans* [12]. The bacterial genes that control the phenotype have not yet been identified—in part owing to the absence of a transformation system for these fastidious intracellular microbes with which to test candidate genes.

*Medea* (maternal effect dominant embryonic arrest) is a cleverly constructed acronym that doubles as a nod to Greek mythology. Medea was the sorceress who helped Jason win the golden fleece, but sadly they did not live happily ever after: he later left her for another princess and so, at least in Euripides' version, she killed their children in bloody revenge. *Medea* elements can likewise cause the death of the progeny of heterozygous females, unless they also carry a *Medea* element, through the expression of an unidentified “toxin” in the germline of *Tribolium* females and an “antidote” in the embryo stage [6],[13]. Like cytoplasmic incompatibility, this provides a powerful frequency-dependent drive that can cause rapid population spread of the element [14]. At least two independently acting *Medea* elements occur at different locations in the *Tribolium* genome [13], and *Medea* has been shown to be associated with a 21-kb composite *Tc1* transposable element insertion [15]. The mechanism of action remains unknown but intriguingly, the *Tc1* element contains a gene that is apparently of bacterial origin, and the insertion is located just downstream of a *Tribolium* gene whose *Drosophila* ortholog (“*blot*”) has both maternal and zygotic functions. Analogous systems have also been reported in mice: *scat* (severe combined anemia and thrombocytopenia), associated with a maternally conferred autoimmune disease [16],[17], and HSR (homogeneously staining region) which impart maternal lethality to late embryos [18]; both can be prevented by zygotic expression of the element if inherited from either parent.

The *peel* (paternal effect epistatic embryonic lethal)—*zeel* (zygotic epistatic embryonic lethal) incompatibility element in *C. elegans* was first reported by Hannah Seidel and colleagues in 2008 [19]. The offspring of males heterozygous for the element will die at the late embryo stage unless they inherit at least one copy, and can thus express the ZEEL-1 “antidote.” The trait was mapped to a 62-kb region that shows an unusual degree of divergence between the “Bristol” *peel-zeel*–containing haplotype and the “Hawaii”-sensitive haplotype. The *zeel-1* “rescue” gene was identified in this interval and encodes a membrane-spanning protein [19]. There are clear parallels with cytoplasmic incompatibility, with which *peel-zeel* shares a paternal mode of “toxin” delivery via modification of sperm (Figure 1). There are also contrasting features—*Wolbachia* and *Cardinium* are intracellular parasites with maternal inheritance that is not always 100%, and can be cured with antibiotics, while *peel-zeel* is a genetic element located in the nuclear genome and subject to the usual laws of Mendelian segregation (as indeed is Medea). Furthermore *peel-zeel*–induced embryo mortality is late acting, while early embryo death is the norm in *Wolbachia*-induced incompatible crosses (although this is not always the case: late death can occur at quite high frequency in certain cytoplasmically incompatible crosses) [4].

**Figure 1:**
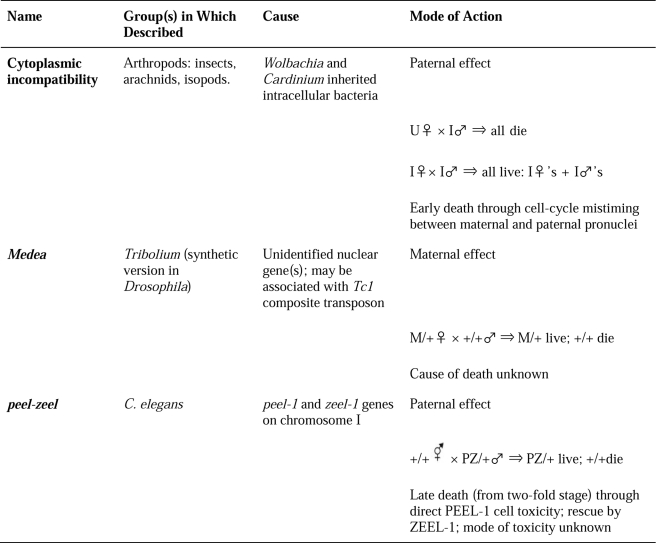
Embryo killer systems in invertebrates. I, infected; U, uninfected.

In this issue of *PLoS Biology*, Seidel et al. [20] have now examined two strains that, unusually, were found to have “rescue” *zeel-1* capacity but no ability to induce paternal-effect embryo killing in the appropriate crosses (analogous to the “mod^−^ resc^+^” strains of *Wolbachia* [21]). By identifying causal mutations they were able to identify a not-previously annotated candidate gene and with an elegant and comprehensive set of further experiments demonstrate beyond doubt that this is indeed *peel-1*. They show that its product—like ZEEL-1 a transmembrane protein—contains a sperm localization signal, is delivered via sperm specific vesicles, and is a highly potent cellular toxin from the embryonic two-fold stage onwards. Muscle and epidermal tissue are particularly affected, and toxicity is still seen in adult tissues. Using impressive single-molecule in situ hybridization techniques the expression of the rescuing *zeel-1* is shown to be tightly controlled in the embryo, and the ZEEL-1 protein efficiently rescues PEEL-1 toxicity [20]. These experiments propel this recently discovered system to the forefront of our mechanistic understanding of invertebrate embryo killing, and demonstrate for the first time that postsegregation distortion can be produced by a comparatively simple binary system—a true toxin and its antidote.

The molecular mechanism of PEEL-1 cellular toxicity is yet to be elucidated, and together with the means of ZEEL-1 rescue, this will be an important area for further research. It is clear though that there are major differences compared to the mode of action of *Wolbachia*. The “toxin” in the latter appears to be a disruption specific to early embryogenesis [8]–[11] rather than a true cellular toxin like PEEL-1 that can also kill cells in other stages and tissues. Sperm lack cytoplasm and thus a straightforward route for mRNA delivery, so paternal effects are far less common in development than maternal effects. The membrane-spanning nature of the PEEL-1 protein may be crucial to its delivery in this respect, and thus to the evolution of the trait. Nevertheless it seems likely that *Medea*-type elements will prove to be more common and widely distributed, given mRNA delivery from a mother to the developing oocyte, than nuclear paternally acting elements like *peel-zeel*. The penetrance of the phenotype in hermaphrodite sperm is incomplete and this is shown to be associated with differences in delivered amount of PEEL-1 toxin according to sperm size [20]. This dosage dependency provides another interesting parallel with *Wolbachia*: cytoplasmic incompatibility between infected males and uninfected females can be incomplete in some species and can be lower in wild males than in the lab [4], associated with reduced densities in the testes. Whether there are any environmental contributions to the expression of *peel*-*zeel* incompatibility, as have been observed for *Wolbachia*, remains to be determined.

The *peel-zeel* region show a paradoxical distribution for a selfish genetic element: it is apparently globally distributed but not at fixation, being present in only about two-thirds of wild isolates of *C. elegans* [19]. The normal expectation is for such elements to go to fixation within populations as a result of their drive, unless suppressor genes or resistant drive targets have arisen; elements are therefore frequently only discovered when crosses between isolated populations, or between sibling species, are undertaken [1]. There is no evidence at all for resistance to/suppression of expression of *peel-1* from the crosses conducted. The high degree of self-fertilization that occurs in *C. elegans* is very likely to underpin the unusual distribution pattern. A high rate of selfing certainly provides a very challenging terrain for a selfish genetic element to invade, given that outcrosses and the resulting heterozygotes are essential for the element to be able to produce its self-favouring phenotype. It seems likely that the *peel-zeel* element evolved, and probably spread to fixation, prior to the transition to the current extreme selfing form of hermaphroditism in this species, which may in fact have been a relatively recent change in evolutionary terms [22],[23]. The haplotypes lacking the element may be maintained in a stable long-term polymorphism, despite its drive, through balancing selection [19],[20]—suggesting there may be mildly deleterious effects of the *peel-zeel* region when homozygous. This could be due to an incomplete rescue of PEEL-1 toxicity by ZEEL-1, or alternatively deleterious effects of linked polymorphisms.

Experiments to tease apart these possibilities are now possible, and data can be used to construct and parameterize mathematical models to examine whether stable polymorphisms of this kind could be maintained (the alternative might be a prediction that a slow-motion increase or decline of the element may actually be in progress). More detailed geographical population studies of *C. elegans* are possible now that both components of the element have been identified. Achieving a better understanding of *C. elegans* outcrossing rates in nature is important. Laboratory population studies, incorporating manipulation of the degree of outcrossing, are also possible given the highly tractable experimental system provided by *C. elegans*.

Because of their powerful population invasion capabilities, both *Wolbachia* and *Medea* have attracted much attention as “drive systems” that could be used to make wild populations of pest insects unable or less able to transmit disease, through natural mechanisms of pathogen inhibition in the case of *Wolbachia* or by spreading linked transgenes in the case of *Medea* [24]–[30]. On the basis of the model of a maternal toxin with a linked zygotic antidote, a synthetic *Medea* element has been created de novo in *D. melanogaster* [31] using maternally expressed microRNAs that silenced a maternally required gene, *myd88*; the antidote was a zygotically expressed variant of *myd88* with a deletion rendering it insensitive to the miRNA. The synthetic element rapidly increased in frequency in population cage experiments, and efforts are underway to create similar systems in mosquito vectors of human disease [31]–[33]. If PEEL-1 would be as toxic to insect cells as it is in *C. elegans*, the *peel-zeel* element could provide a powerful new gene drive system for insect pests. To paraphrase an old saying, one taxon's poison might be another's meat—despite its potency the toxicity might be rather specific—and crucially it would need to be delivered by but not negatively affect sperm, or indeed the developing embryo until after the point at which zygotic genes are expressed. ZEEL-1 seems to completely rescue PEEL-1 toxicity only as the concentrations of the latter are low [20], so achieving appropriate expression (low levels and with tight temporal control) in a new host would be crucial.

Thus the *peel*-*zeel* discoveries reported by Seidel et al. provide a new category of postsegregation distorter, bridging previously known systems, and an already very impressive understanding of how the system works. Studies that shed further light on the population biology and likely mode of evolution of this element, together with biochemical studies of the mode of toxicity of PEEL-1, should prove fascinating. It is also to be hoped that understanding of the means of embryo killing in the insect counterpart systems will advance at a similar rapid rate, allowing more informed comparisons of exactly how these very disparate systems have converged on such a successful strategy of postmeiotic distortion.
